# Low phytic acid pea supplementation as an approach to combating iron deficiency in female runners: A randomized control trial

**DOI:** 10.1177/02601060231181605

**Published:** 2023-06-08

**Authors:** Keely A. Shaw, Philip D. Chilibeck, Donna L. Lindsay, Thomas D. Warkentin, Jongbum Ko, Gordon A. Zello

**Affiliations:** 1 College of Kinesiology, 7235 University of Saskatchewan, Saskatoon, SK, Canada; 2 Crop Development Centre, 7235 University of Saskatchewan, Saskatoon, SK, Canada; 3 Department of Plant Sciences, 7235 University of Saskatchewan, Saskatoon, SK, Canada; 4 College of Pharmacy and Nutrition, 7235 University of Saskatchewan, Saskatoon, SK, Canada

**Keywords:** Ferritin, plant-based nutrition, phytate

## Abstract

**Background:** Iron deficiency (ID) is the most prevalent micronutrient deficiency in the world and the leading cause of anemia globally. Female athletes are at a disproportionate risk for ID due to blood loss through menstruation and decreased iron absorption secondary to exercise. Field peas are a rich source of iron but, similar to iron from other plant-based sources, the iron has limited bioavailability due to high levels of phytic acid, an inherent compound that binds to cations, creating a salt (phytate), which limits absorption during digestion. **Aim:** The purpose of our research was to investigate the effect of a field pea variety bred to have low levels of phytic acid on plasma ferritin, exercise performance, and body composition in female runners. **Methods:** Twenty-eight female runners (age:34.6  ±  9.7 years; weight: 65.1  ±  8.1 kg; VO_2_max: 50.7  ±  8.9 ml/kg/min) underwent measures of ferritin, exercise performance, and body composition before and after being randomly assigned to consume a powder derived from regular peas, low phytic acid peas, or a non-pea control (maltodextrin), plus vitamin C for 8 weeks. **Results:** The regular pea and low phytic acid pea groups had a 14.4% and 5.1% increase in plasma ferritin, respectively, while the maltodextrin group had a decrease of 2.2%; however, the difference in changes between groups was not statistically significant. No differences between groups were evident in any of the other measures. **Conclusion:** Larger doses or longer duration of pea supplementation may be necessary to induce meaningful changes in iron status. This trial was registered at ClinicalTrials.gov (NCT04872140).

## Introduction

Iron plays a crucial role in human physiology such as oxygen transport, energy production, and cell proliferation (Shoemaker et al., 2019). Despite the importance of iron, iron deficiency (ID) is the most prevalent micronutrient deficiency globally, with athletic individuals being impacted to a greater extent than their sedentary peers. Research suggests 15–35% of female athletes and 3–11% of male athletes present with ID, though prevalence of >50% in females and up to 30% in males have been reported (Sim et al., 2019; Sinclair and Hinton, 2005). Increased prevalence of ID in females is related to blood loss through menstruation (Mirza et al., 2018; Pasricha et al., 2014), further exacerbated in athletes due to exercise-induced losses through foot strike hemolysis, ischemia to the viscera, frequent use of anti-inflammatory drugs, or increased losses through urine and sweat (Coates et al., 2017). Further, the requirement for iron may be increased due to increased erythropoiesis in response to aerobic exercise (Clénin et al., 2015).

In addition to the fatigue generally observed in ID, athletes with ID are likely to experience reduced performance, training tolerance, and recovery from training (McCormick et al., 2020). To maximize their iron levels and performance, athletes often turn to pharmaceutical interventions. Skorseth et al. (2020) reported that 42% of high school distance runners report taking an iron supplement, while Coates et al. (2017) reported significant intakes of supplemental iron in elite runners and triathletes. Pharmaceutical interventions appear beneficial for improving serum ferritin levels (Pasricha et al., 2021), though they are reported to cause gastrointestinal disturbances (Sim et al., 2019).

When considering dietary approaches to combat ID, the literature is limited and inconclusive. Some research has reported increases in serum ferritin after increasing dietary iron intake (Ishizaki et al., 2006) while others have found no change in ferritin levels with the inclusion of 255 g/week of lean beef (Burke et al., 2012) or diets designed to deliver 18.5 mg of dietary iron daily (Alaunyte et al., 2014).

Field peas are a rich source of macro- and micro-nutrients, including iron (Bangar et al., 2017). However plant-based proteins are also high in phytic acid. This antinutritional compound binds to cations such as iron and zinc, forming a salt (phytate) that is then excreted, decreasing the bioavailability of the mineral (Warkentin et al., 2012). The Crop Development Centre at the University of Saskatchewan developed two lines of field peas with low phytic acid (*lpa*) through chemical mutagenesis (Warkentin et al., 2012), a cost-effective plant breeding technique used to optimize nutrient status (Bouis et al., 2011). These *lpa* lines have approximately 60% less phytic acid while performing similarly agronomically (Lindsay et al., 2021) and have 1.4 and 1.9 times higher iron bioavailability than the parent lines (Liu et al., 2015). These *lpa* lines may potentially be used in a plant-based dietary approach to combat ID in affected individuals. The current study aims to assess the effectiveness of a powder made from *lpa* peas for improving iron status and exercise performance in female runners. We hypothesized that the *lpa* peas would improve iron status (i.e., ferritin) to a greater extent than the regular-phytic acid peas or a non-pea control which may, in turn, improve exercise performance.

## Methods

### Participants and Study Design

This study utilized a randomized, double-blind, placebo-controlled parallel-groups design and was approved by the Biomedical Research Ethics Board at the University of Saskatchewan (BIO 1207). Sample size calculations were conducted using data from Anschuetz et al. (2010), assuming that our pea product would change dietary iron bioavailability from low bioavailability to medium-high bioavailability. Based on an effect size of 1.61 (Anschuetz et al. (2010) while using an alpha of 0.05 and 80% power, calculations suggested a sample size of nine participants per group to be sufficient. A convenience sample of 34 women was recruited from the Saskatoon, Saskatchewan area from September 2021 to January 2022 to take part in the research. Inclusionary criteria included being a regular runner (≥2 days per week, ≥20 min each time), not having supplemented with pharmaceutical iron in the past 6 weeks, not postmenopausal, and having an intake of dietary iron below the recommended daily allowance (RDA) of 18 mg per day (Institute of Medicine, 2001). Participants were excluded if they were deemed to be anemic based on hemoglobin analysis (<115 g/L). After providing written free and informed consent, participants completed a 3-day food diary. If iron intakes were below the RDA, participants underwent baseline testing sessions, followed by randomization to one of three dietary supplements (described below). After the 8-week supplementation period, participants completed post-testing, which was identical to baseline testing but without a familiarization time trial. Randomization was completed in blocks of three by a researcher not involved in any other part of the study. The allocation schedule was concealed from the researcher recruiting participants. All baseline and post-testing measurements were conducted at the same time of day (± 60 min). This trial was registered at ClinicalTrials.gov (NCT04872140).

### Dietary Intervention

Participants were randomly assigned by a computer random-number generator to consume one of three powders for 8 weeks: a powder made from regular peas, a powder made from peas bred to have *lpa*, or a non-pea control (maltodextrin). Intervention length was based on reports of 4–8 weeks of supplementation to produce an increase in serum ferritin (Blee et al., 1999), as well as intervention lengths in other food-based approaches to improve iron status of 4–8 weeks (Alaunyte et al., 2014; Anschuetz et al., 2010; Burke et al., 2012). Participant randomization was sent to a third-party researcher with no other role in the study, who prepared the powder. Participants and the researcher responsible for data collection and analysis were blinded to intervention assignment. Nutritional information of dietary conditions is displayed in Table 1. Pea powders were equated for iron delivered through the supplement. The *lpa* pea contained slightly more iron than the regular pea (51.4 vs. 47.9 mg/kg), so those randomized to the regular pea group consumed slightly more supplements daily. The maltodextrin condition was approximately equated for calories. Participants consumed the daily amount broken up into three doses spread throughout the day in the way that best fit their lives and taste palettes but were instructed not to consume the supplement within 2 h of any dairy or dairy alternatives to optimize iron absorption. Participants were given no other instruction on their dietary intake but were informed of the approximate caloric load of the supplement and instructed to adjust their diet in order to maintain their habitual energy intake. Participants were also provided 125 mg tablets of vitamin C, which they were instructed to consume alongside each dose of supplement in order to enhance iron absorption. Each participant recorded compliance in a log and leftover powder was collected and weighed to confirm.

**Table 1.** Nutritional information of supplements.

	Low phytic acid pea	Regular pea	Maltodextrin
Daily amount (g)	120	130	125
Energy (kcal)	498	540	500
Carbohydrate (g)	53.2	57.6	125
Protein (g)	58.8	63.7	0
Fat (g)	5.6	6.1	0
Iron (mg)	6.2	6.2	0.1

### 3-Day Food Diary

Regular dietary intake was assessed using a 3-day food diary. Participants were instructed to record their food and beverage intake on 3 days, including one weekend day. Participants were instructed not to record on days they did not consider to be “normal” (they were ill, went to a celebration, etc.) and to provide as much detail for each food item as possible, including product brands where applicable. Dietary intakes from these 3 days were assessed using Chronometer (https://cronometer.com), with confirmation of nutritional data performed through cross-referencing with the Canadian Nutrient File (2015) where necessary. Inadequate dietary iron intakes relative to the RDA were used as a proxy for iron status, therefore individuals with dietary iron consumption below the RDA of 18 mg/day were invited to complete baseline testing.

### Body Composition

Participants arrived at the research facility in a normally fed and hydrated state and were instructed to avoid vigorous exercise in the 24 h prior to the visit. Body composition was measured by dual-energy X-ray absorptiometry (Hologic© Discovery Wi; Bedford, MA) using QDR software for Windows XP (QDR Discovery, Hologic, Inc.). Measurements were obtained by a certified radiology technologist. Weight and height were assessed using a calibrated weigh scale and stadiometer, respectively. Reproducibility of repeated measures of lean tissue mass and fat mass in our facility have coefficients of variation of 1% and 3%, respectively.

### Hematology

A venous blood sample was collected by a trained phlebotomist and then left for 10–60 min at room temperature before being centrifuged at 3000 *g* for 10 min at 10°C. Plasma was then separated into microcentrifuge tubes and stored at −80°C until analysis. When all plasma samples were collected (pre- and post-intervention), samples were transferred to a clinical laboratory (Royal University Hospital, Saskatoon) for analysis of ferritin and C-reactive protein, which were assessed using electrochemiluminescence immunoassay and particle enhanced immunoturbidimetric assay, respectively. Two trials of all plasma measures were run and results were averaged for data analyses. Hemoglobin was determined by a fingertip using capillary samples and assessed by a point-of-care testing device (HemoCue, Ängelholm, Sweden). Participants with hemoglobin levels <115 g/L were excluded from the study and referred to their healthcare practitioner due to risk of anemia.

### Maximal Oxygen Consumption (VO_2_max)

Participants were instructed to follow their typical pre-exercise nutrition, hydration, and caffeine intake and arrive to the research facility having not engaged in moderate to vigorous exercise in the 24 h prior. Upon arrival, participants rested for 5 min, after which heart rate and blood pressure measures were taken. Following resting measures, participants completed a standardized 5-min treadmill warmup, during which they walked for 3 min at a speed they considered a “comfortable warmup walk” and ran for 2 min at what they thought to be a pace they could hold for 20 min but not longer. These speeds were recorded and replicated for post-intervention assessments. Participants were then given a 5-min rest period while a heart rate monitor strap and gas collection mask were fitted. Participants underwent a progressive exercise test in which they ran at the speed chosen in the warmup and the grade of the treadmill increased by 2% every 2 min until volitional fatigue. Expired gases (Vmax Series 29 Calorimeter, SensorMedics, USA) and heart rate (Polar, Kempele, Finland) data were collected for the test duration and averaged over 20-s. Participants were considered to have reached VO_2max_ if there was a plateau in oxygen consumption with increasing work rate and a respiratory exchange ratio of >1.1. If these criteria were not met, participants were considered to have reached VO_2peak._ After an active cooldown of at least 5 min, participants rested passively for 30 min before completing a familiarization 5 km time trial, as below.

### 5 km Time Trial

At least 36 h later, participants arrived at our facility having followed the same pretest instructions as for the maximal oxygen uptake test. Participants completed a 500 m warmup walk at the pace chosen during the first 3 min of the warmup in the previous exercise session and at a 1% grade incline. Immediately upon completion of the 500 m, participants were given control of the treadmill speed and instructed to complete the 5 km as quickly as possible. Percent grade of the treadmill remained at 1% for the duration of the run. Participants were permitted to see the treadmill speed and distance covered but were blinded to the time elapsed. Expired gases and heart rate were collected continuously during the warmup and 5 km run. Lactate measurements were assessed using fingertip capillary samples (Lactate Scout; EKF Diagnostics, Wales, United Kingdom) at rest as well as every 5 min during the 5 km run. Because test durations differed between participants, lactate, heart rate, and expired gas data were normalized to six-time points (i.e., warm-up (or rest for lactate), quintiles of the 5 km run).

### Biweekly Check-ins

Participants were instructed to maintain their habitual physical activity and dietary behaviors, and were asked to try to maintain their pre-intervention energy intake during the intervention period (i.e., to replace a dessert or snack with the supplement so as to not increase caloric intake due to the supplement). Physical activity and dietary habits were monitored through questionnaires sent every 2 weeks. A link over email every 2 weeks to complete a questionnaire delivered using the online platform SurveyMonkey. In this questionnaire, participants recorded a 24-h food log and completed the Godin Leisure Time Exercise Questionnaire (Godin and Shephard, 1985). Participants were also given an opportunity to report any symptoms or side effects they were experiencing that they thought might be related to the supplement. Participants were provided with examples of symptoms they might experience (bloating, decreased appetite, increased flatulence, etc.), but encouraged to report any symptom they thought might be related to consumption of the supplement. Total dietary intakes (regular diet plus supplement) were assessed using an online assessment tool, Chronometer, as described above.

### Statistics

All data were analyzed using JASP statistical software (version 16.2; 2021, University of Amsterdam). Data are expressed as mean  ±  standard deviation unless otherwise stated. Prior to analysis, data were assessed for normality through assessment of skewness and kurtosis and outliers were assessed using boxplots. Data were considered to be outliers if they exceeded 1.5 times the interquartile range of the boxplot. Body composition, 5 km time trial performance, hemoglobin, ferritin, C-reactive protein, and maximal oxygen uptake were assessed using a 3  ×  2 (group × time) mixed ANOVA, with repeated measures on the “time” factor. Measures during the 5 km time trial were equated to six-time points to correct for different duration of the test between participants: warmup (or rest for lactate) and then quintiles during the run. Lactate, heart rate, and expired gas data from the 5 km time trial were assessed using a 3  ×  2  ×  6 (group × time [pre-/post-intervention] × time point during the run) ANOVA, with repeated measures on the last two factors. Tukey's LSD post hoc tests were used to compare means if any main effects or interactions were observed. Frequency of symptoms between groups was assessed using chi-square tests. Effect size for change scores between conditions was calculated using partial η^2^ (η_p_^2^) for our primary (ferritin) and secondary (exercise variables; VO_2_max and 5 km time trial) outcomes. An effect size of 0.01 was considered small, 0.06 as medium, and 0.14 as large. Significance was accepted at *p* < 0.05.

## Results

**Figure 1.**
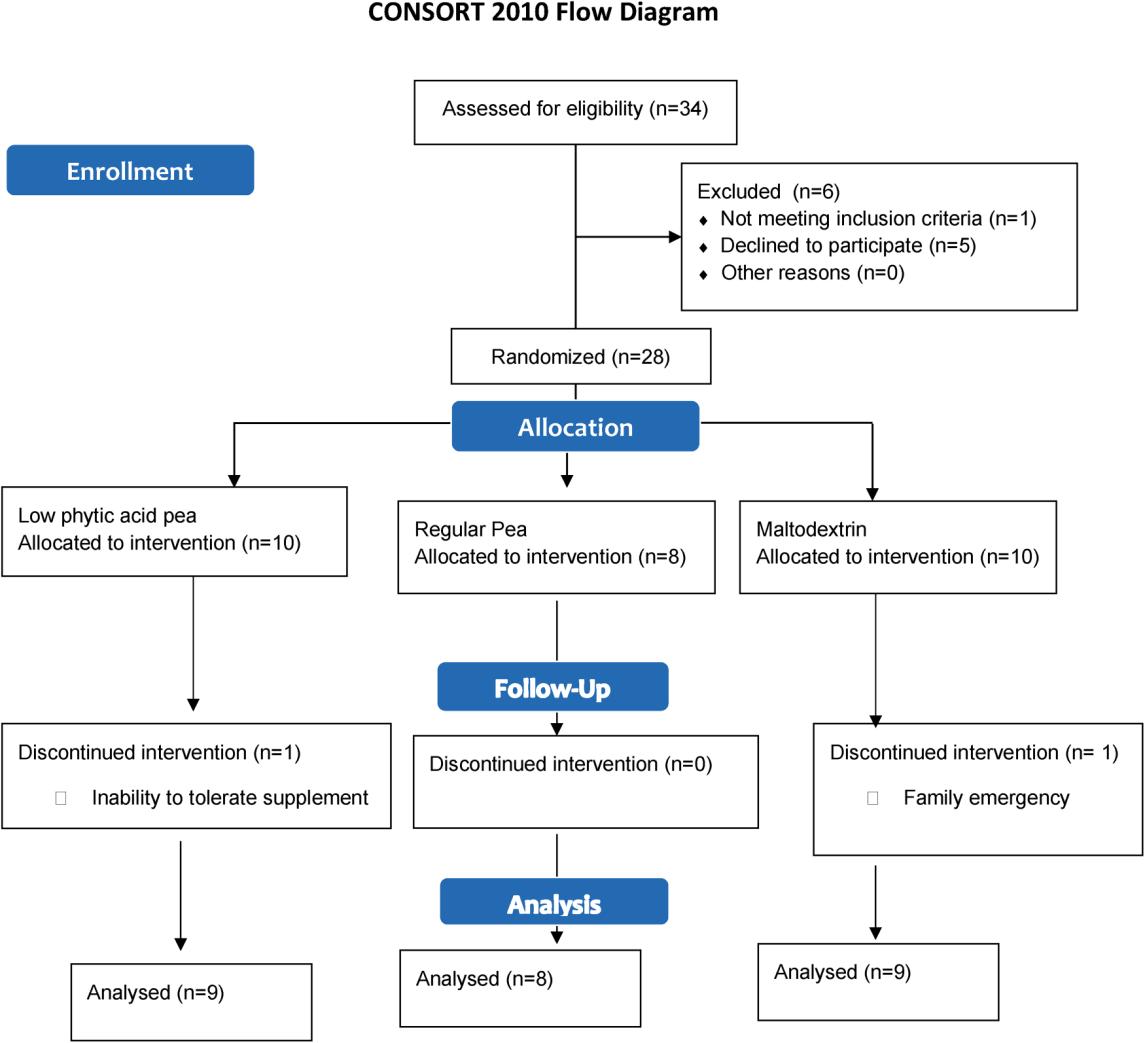
Flow diagram of participant recruitment.

Thirty-four women consented to take part in the study (Figure 1). Twenty-eight participants were randomized and began the supplement. One dropped out due to an inability to tolerate the supplement, and another due to reasons unrelated to the study. The final analysis included nine individuals from each the maltodextrin and regular pea groups and eight from the *lpa* group. Demographic information is presented in Table 2. No differences were observed between groups at baseline.

**Table 2.** Participant demographic information.

	Maltodextrin	Regular pea	Low phytic acid pea
Age (years)	33.7 ± 8.6	35.1 ± 13.1	34.9 ± 8.6
Weight (kg)	69.3 ± 8.2	62.3 ± 8.6	65.3 ± 6.9
VO_2_max (ml/kg/min)	47.8 ± 8.7	51.6 ± 9.9	53.4 ± 8.4
5 km time (min)	30.8 ± 4.1	27.7 ± 2.4	28.5 ± 3.3
Running frequency (days/week)	3.2 ± 1.0	3.9 ± 1.4	3.6 ± 1.3
Running volume (min/week)	134 ± 67	152 ± 58	173 ± 103

All values are means  ±  SD. VO_2_max  =  maximal oxygen consumption.

### Compliance

Compliance was greater than 80% in all groups (maltodextrin  =  81.9%; regular pea  =  85.5%; *lpa* pea  =  82.6%). One participant in the maltodextrin group consumed 1/5 of the prescribed dose and one consumed 2/3 of the prescribed dose. One participant in the *lpa* pea group consumed 2/3 of the dose, one 3/4 of the dose, and another consumed half the dose. Two participants in the regular pea group consumed 2/3 of the dose. All other participants consumed the supplement as prescribed.

### Hematological Measures

**Figure 2.**
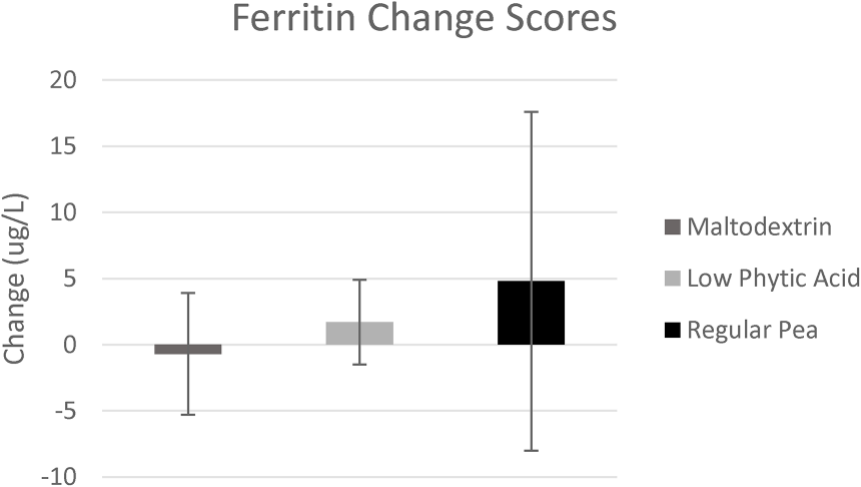
Change in ferritin values (ug/L) after the 8-week dietary intervention. Error bars represent standard error of the mean.

*Ferritin*. Changes in ferritin levels between the beginning and end of the trial are depicted in Figure 2. Before analysis, two outliers were removed from the maltodextrin group, two from the regular pea group, and one from the *lpa* group. No group × time interaction was evident (*p*  =  0.75; η_p_^2^  =  0.014; Table 3). One participant had high C-reactive protein levels at baseline, so a sensitivity analysis was performed with that datum removed, as high inflammation can affect iron status. This did not alter the findings. Further sensitivity analysis was conducted looking at only those participants below the median ferritin value at baseline, with no differences evident in the results. Note that inclusion of outliers in the analysis did not alter the findings. Further, findings were not altered if compliance to the supplement was added as a covariant nor if ferritin data were log-transformed.

**Table 3.** Outcome measures at baseline and post-intervention for each group.

Measure	Time	Maltodextrin	Low phytic acid pea	Regular pea
Ferritin (ug/L)	Pre	31.3 ± 12.2	33.4 ± 19.2	33.4 ± 22.6
	Post	30.6 ± 19.4	35.1 ± 25.6	38.2 ± 27.5
CRP(mg/L)	Pre	0.66 ± 0.66	0.49 ± 0.36	0.46 ± 0.15
	Post	0.67 ± 0.56	0.51 ± 0.24	0.73.46
Hemoglobin (g/L)	Pre	130.8 ± 9.3	132.3 ± 10.8	129.8 ± 9.1
	Post	131.4 ± 8.3	130.4 ± 10.3	133.4 ± 9.9
VO_2_max (mL/kg/min)	Pre	49.7 ± 7.5	53.4 ± 8.4	53.6 ± 8.8
	Post*	46.8 ± 2.7	48.6 ± 4.8	47.0 ± 3.6
VO_2_max (L/min)	Pre	3.4 ± 0.9	3.5 ± 0.6	3.2 ± 0.4
	Post*	3.3 ± 0.6	3.2 ± 0.6	2.9 ± 0.4
5 km time trial (s)	Pre	1850.8 ± 246.3	1725.7 ± 210.8	1660.4 ± 142.8
	Post	1848.6 ± 257.6	1722.7 ± 204.8	1651.2 ± 166.7
Fat mass (kg)	Pre	17.7 ± 5.8	14.8 ± 4.6	16.5 ± 5.3
	Post	17.7 ± 5.2	15.3 ± 4.9	16.4 ± 3.9
Lean mass (g)	Pre	48.5 ± 5.4	47.7 ± 4.7	43.1 ± 3.5
	Post*	49.4 ± 4.5	47.9 ± 4.6	44.5 ± 3.3

Values are mean and SD. CRP  =  C-reactive protein; VO_2_max  =  maximal oxygen consumption.

*Time main effect (values across groups during post-testing different from baseline; *p* < 0.05).

C-reactive protein and hemoglobin. One outlier from the maltodextrin group was removed prior to analysis for C-reactive protein. There was no interaction between group and time for C-reactive protein (*p*  =  0.22) or hemoglobin (*p*  =  0.31; Table 3).

### Exercise Performance

VO_2_max. No significant interaction was found between group and time (*p*  =  0.64; η_p_^2^  =  0.05). There was a significant time effect, with baseline VO_2_max values significantly higher than post-intervention (*p*  =  0.005). One participant in the *lpa* group became very ill with COVID-19 and mononucleosis approximately midway through the dietary intervention, impacting her training and fitness. Removal of her data had no impact on the findings (Table 3).

5 km time trial. One outlier from the maltodextrin group was removed prior to analysis. No interaction was found between group and time (*p*  =  0.55; η_p_^2^  =  0.06; Table 3). When the participant who became ill during the intervention was removed, the results were unchanged. During the 5 km run, there was no group × time or group × time × time during the run interactions for oxygen consumption, lactate, respiratory exchange ratio, or heart rate (*p* > 0.05; Table 3). As expected, there were main effects of time evident during the run (*p* < 0.05), with heart rate being significantly lower during the warmup than during the run, VO_2_ increasing from warmup to conclusion of the run, and lactate being lowest at rest and highest at the last collection point (i.e., near the end of the run).

### Body Composition

One participant from the maltodextrin group did not undergo baseline body composition assessment due to scheduling issues. No interactions were found between group and time for lean body mass, fat mass, or percent fat (*p* > 0.05). There was an effect of time for lean mass (*p*  =  0.009), with post-intervention assessments being significantly greater than those obtained at baseline (46.5  ±  5.0 kg vs. 47.3  ±  4.5 kg).

### Biweekly Check-ins

Dietary intakes from both regular dietary intake and the supplement are portrayed in Table 4. No differences were observed in energy (*p*  =  0.55), carbohydrate (*p*  =  0.41), or fat intake (*p*  =  0.20) throughout the trial. Both protein (*p*  =  0.03) and iron (*p*  =  0.01) intake were significantly greater in the regular pea group compared to the maltodextrin group. No other differences in dietary intake were observed. Leisure time exercise questionnaire responses did not differ between groups nor across time points (*p* = 0.69; data not shown).

**Table 4.** Dietary intake at baseline and every two week during the trial

	Baseline	Week 2	Week 4	Week 6	Week 8
Energy (kcal/day)					
Maltodextrin	1849 ± 445	2181 ± 320	2194 ± 494	1916 ± 421	2202 ± 453
Low phytic acid	2078 ± 297	1990 ± 272	1966 ± 316	2053 ± 412	2172 ± 599
Regular pea	1762 ± 424	2177 ± 317	1839 ± 529	1816 ± 246	2074 ± 584
Carbohydrates (g/day)					
Maltodextrin	202.4 ± 60.3	287.5 ± 60.6	239.0 ± 27.8	236.0 ± 54.0	266.7 ± 70.4
Low phytic acid	245.2 ± 69.4	205.1 ± 58.5	213.0 ± 38.4	232.3 ± 69.9	230.2 ± 95.9
Regular pea	200.1 ± 69.8	249.5 ± 74.0	219.5 ± 42.2	215.0 ± 45.0	227.8 ± 85.5
Protein (g/day)					
Maltodextrin	97.2 ± 41.3	80.7 ± 28.6	90.7 ± 25.8	88.4 ± 32.6	86.0 ± 32.7
Low phytic acid	86.5 ± 20.8	124.8 ± 36.4	104.8 ± 23.4	119.4 ± 24.9	117.4 ± 38.9
Regular pea*	64.9 ± 12.5	111.3 ± 25.9	106.3 ± 21.8	125.8 ± 31.1	114.7 ± 31.4
Fat (g/day)					
Maltodextrin	72.9 ± 19.5	64.6 ± 20.2	61.0 ± 26.9	63.1 ± 19.6	80.4 ± 36.5
Low phytic acid	83.2 ± 13.1	86.0 ± 22.0	68.7 ± 23.4	78.8 ± 16.8	71.6 ± 26.7
Regular pea	79.9 ± 33.2	63.2 ± 25.5	55.5 ± 25.4	55.4 ± 14.8	71.5 ± 22.8
Iron (mg/day)					
Maltodextrin	12.7 ± 3.8	12.3 ± 4.4	12.6 ± 7.1	11.3 ± 7.3	11.6 ± 2.4
Low phytic acid	13.7 ± 1.8	16.7 ± 2.6	15.0 ± 3.6	16.4 ± 4.4	14.3 ± 4.7
Regular pea*	11.2 ± 3.8	18.2 ± 6.6	17.1 ± 5.8	15.2 ± 2.9	16.3 ± 5.3

Note: Values are means and SD. Values include the regular diet combined with the pea or placebo supplementation.

*Simple main effect of condition was evident for protein and iron intake throughout the intervention, with higher intakes in the regular pea compared to the maltodextrin group (*p* < 0.05).

### Adverse Effects

Approximately 40% of participants reported some variety of symptoms they believed to be related to the supplement during the trial, with more symptoms being reported from the pea groups than the maltodextrin group for the first three check-ins. No statistical differences were observed at week 2 (χ^2^  =  2.841, *p*  =  0.24), week 6 (χ^2^  =  3.646, *p* =  0.16), or week 8 (χ^2^  =  0.572, *p*  =  0.75) for the prevalence of symptoms. A significant difference in the prevalence of symptoms was observed at week 4, with the pea conditions experiencing more symptoms than the maltodextrin group (χ^2^  =  6.766, *p*  =  0.03). Zero to two individuals reported symptoms from the maltodextrin group compared to three to six participants from the pea groups. At the final check-in, the number of participants that reported symptoms were similar between groups. The most common symptom reported was bloating, followed by gassiness. Other reported symptoms included abdominal pain, constipation, heartburn, decreased appetite, nausea, and diarrhea, though these were less common. The prevalence of symptoms was highest at the first check-in and decreased until the third check-in, at which time they remained stable to the end of the trial.

## Discussion

This research is the first of its kind to investigate the role of a plant-based dietary intervention using peas with increased iron bioavailability on iron levels in female runners. No statistical differences in plasma ferritin levels were observed between the groups, though both pea groups experienced a net increase in serum ferritin, whereas the maltodextrin group did not.

The current findings corroborate other literature investigating food-based approaches ID in athletes. Alaunyte et al. (2014) failed to observe differences in serum ferritin in deficient females consuming 7 mg/day of iron in the form of an iron-fortified bread for 6 weeks. Similarly, Burke et al. (2012) reported no significant change in plasma ferritin after both male and female cross-country runners consumed 9 oz red meat per week plus a multivitamin supplement. Burke et al. (2012) did, however, report a non-significant increase in hematocrit. Given that Burke et al. (2012) utilized a pharmaceutical supplement plus animal-sourced iron (i.e., heme iron from red meat), the validity of comparison to the current trial is limited, as the absorption of heme and non-heme iron is substantially different (Piskin et al., 2022; Shubham et al., 2020). Anschuetz et al. (2010) also failed to elicit a significant change in iron status after a 4-week intervention in collegiate runners who received dietary counseling to improve iron absorption. Despite lacking statistical significance, the authors noted that individuals who improved the bioavailability of iron in their diet experienced increases in serum iron parameters.

The results of the current study differ from observations by Ishizaki et al. (2006), who found improved serum ferritin in gymnasts after 4 weeks of consuming diets designed by a dietitian to provide 15 mg of iron per day through a traditional Japanese diet, though benefits disappeared 4 weeks after stopping the dietary intervention. Differences observed between studies could be due to a myriad of factors, including the type of iron investigated (heme, non-heme, or a combination), as well as iron levels at baseline. It is well-established that absorption of dietary iron is inversely related to iron status in the body (Kalasuramath et al., 2013), primarily due to the actions of hepcidin (Viatte and Vaulont, 2009). The subjects of the current study were not in a state of iron depletion, with average baseline ferritin values (after removing outliers) of 32.7 ug/L. Despite previous reports failing to detect statistical significance, as mentioned above, net increases in serum ferritin have been recorded. Alaunyte et al. (2014) observed a 5.4% increase in serum ferritin over a 6-week trial of consuming an iron-fortified bread but did not include a control group for comparison. Burke et al. (2012) observed a 15% increase in serum ferritin in the intervention group compared to a 10% increase in the control group, though this research was not a plant-based approach. Future studies investigating plant-based approaches to improve iron status in athletes with larger sample sizes and/or longer intervention periods are warranted, given the positive findings in both Alaunyte et al. (2014) and the current research, the only two studies known to the authors that explore a plant-based approach to increase ferritin levels.

Supplementing with pea protein increased intake of iron and protein in those consuming the regular pea protein, but not the *lpa* pea protein. Differences in protein intake between the two pea groups can be explained by the regular pea group consuming slightly more supplement than the *lpa* pea group, and therefore more protein daily (63.7 vs. 58.8 g/day). However, the two pea powders were matched for iron such that both conditions consumed ∼6 mg/d of extra iron through the pea powder daily. The regular pea group did report slightly better compliance to the supplement than the *lpa* pea group (85.5% vs. 82.6%), which could explain the differences between these two groups. To our knowledge, no research has investigated the feasibility of a pea protein in improving dietary intakes of iron or protein, though research has reported pea protein to perform similarly to whey in inducing muscle hypertrophy and strength (Babault et al., 2015; Banaszek et al., 2019). Pea protein may therefore be an attractive option to those wishing to supplement protein but wishing to limit consumption of animal-based proteins. A pea protein concentrate may be especially attractive to endurance athletes due to the relatively high carbohydrate content (∼39% carbohydrate), which would assist athletes in attaining both their protein and carbohydrate goals (Thomas et al., 2016).

Our study is strengthened by measuring C-reactive protein in addition to serum ferritin, allowing us to eliminate inflammation as a confounding factor on ferritin values (Dignass et al., 2018). However, our study was limited by the wide range of ferritin values of participants. Dietary iron intake was used as a proxy of iron status at baseline, assuming those with low dietary iron intakes would be more likely to have low iron stores. However, some participants presented with very high serum ferritin levels at baseline even after outliers were removed. Future research should investigate the role of pea protein, both regular and *lpa*, on the iron status of individuals with depleted ferritin at baseline. Further, studies on those iron deficient (measured using ferritin status) as opposed to iron inadequate (determined by intake in our study) would be prudent, as those with sufficient ferritin are unlikely to respond to an intervention aimed at improving ferritin.

While much of the current literature conducted to improve iron status in women of reproductive age utilize pharmaceutical iron supplements, delivering a similar amount of iron through a pea product is likely infeasible. However, peas may be more cost-effective while providing other nutritional benefits and beig culturally appropriate in many area of the world most affected by ID. Therefore, future research should investigate the influence of replacing regular peas with *lpa* peas in the diet of those in areas of the world most impacted by ID, especially those in lower-income countries with a high reliance on plant-based diets (Safiri et al., 2021). Such research should consider longer supplementation periods to counteract the lower amount of iron delivered by the peas.

The timing of our testing may also be a limiting factor, as baseline testing happened mostly in October and November, with post-intervention assessments occurring largely in December and January. This may help to explain the main effect of time displaying a decrease in VO_2_ max values from baseline to post-interventions across groups, given the cold climate at the site of the intervention during the winter months (December and January). While physical activity was measured using the Goodin Leisure Time Exercise Questionnaire, it does not account for specific types of activities engaged in. It is possible that participants decreased their frequency of running and increased it with other varieties of exercise during the colder months, which could explain this change.

In conclusion, supplementation with *lpa* peas did not significantly impact ferritin in female runners, though further investigation with tight inclusionary criteria is warranted. Although the results of the current study are not statistically significant, the groups consuming the regular and *lpa* pea powders experienced a small positive effect on plasma ferritin while the maltodextrin group did not. Given the current trend toward reducing the consumption of animal products (Iwasa-Madge and Wegener, 2020; Meyer et al., 2020), pea protein is a feasible option to help athletes meet their protein goals. Further research, potentially with more participants and a longer study period, is needed to clarify whether pea-based supplements could be a strategy for improving iron status.
